# Renal Function, Bisphenol A, and Alkylphenols: Results from the National Health and Nutrition Examination Survey (NHANES 2003–2006)

**DOI:** 10.1289/ehp.1002572

**Published:** 2010-12-08

**Authors:** Li You, Xiangzhu Zhu, Martha J. Shrubsole, Hong Fan, Jing Chen, Jie Dong, Chuan-Ming Hao, Qi Dai

**Affiliations:** 1 Division of Nephrology, Huashan Hospital and; 2 Institute of Nephrology, Fudan University, Shanghai, China; 3 Department of Medicine, Vanderbilt Epidemiology Center, Vanderbilt University School of Medicine, Nashville, Tennessee, USA; 4 Department of Medicine/Renal Division, Peking University First Hospital, and Institute of Nephrology, Peking University, Beijing, China; 5 Department of Medicine/Division of Nephrology, Vanderbilt University, Nashville, Tennessee, USA

**Keywords:** APs, BPA, NHANES, renal function, urinary excretion

## Abstract

**Background:**

Urinary excretion of bisphenol A (BPA) and alkylphenols (APs) was used as a biomarker in most previous studies, but no study has investigated whether urinary excretion of these environmental phenols differed by renal function.

**Objective:**

We estimated the association between renal function and urinary excretion of BPA and APs.

**Methods:**

Analyses were conducted using data from the National Health and Nutrition Examination Survey (NHANES) 2003–2006. Renal function was measured as estimated glomerular filtration rate (eGFR) calculated by the Modification of Diet in Renal Disease (MDRD) Study equation and by the newly developed Chronic Kidney Disease Epidemiology Collaboration (CKD-EPI) equation. Regression models were used to calculate geometric means of urinary BPA and APs excretion by eGFR category (≥ 90, 60–90, < 60 mL/min/m^2^) after adjusting for potential confounding factors.

**Results:**

When we used the MDRD Study equation, participants without known renal disease (*n* = 2,573), 58.2% (*n* = 1,499) had mildly decreased renal function or undiagnosed chronic kidney disease. The adjusted geometric means for urinary BPA excretion decreased with decreasing levels of eGFR (*p* for trend = 0.04). The associations appeared primarily in females (*p* for trend = 0.03). Urinary triclosan excretion decreased with decreasing levels of eGFR (*p* for trend < 0.01) for both males and females, and the association primarily appeared in participants < 65 years of age. The association between BPA and eGFR was nonsignificant when we used the CKD-EPI equation.

**Conclusions:**

Urinary excretion of triclosan, and possibly BPA, decreased with decreasing renal function. The associations might differ by age or sex. Further studies are necessary to replicate our results and understand the mechanism.

Bisphenol A (BPA), a synthetic estrogen, and alkylphenols (APs) are widely used industrial chemicals that have received tremendous media and research attention in recent years because of their estrogenic endocrine-disrupting properties (Turner and Sharpe 1997). Humans are exposed to these environmental phenolic compounds through foods and beverages as well as air, drinking water, dust, soil, and personal care products (Vandenberg et al. 2007). BPA and APs [including 4-*tert*-octylphenol (tOP), benzophenone-3 (BP-3), and the chlorophenol triclosan] may rapidly be cleared by the kidneys and excreted in urine (Dekant and Völkel 2008; Kadry et al. 1995; Kawaguchi et al. 2004; Sandborgh-Englund et al. 2006). By measuring urinary levels of these compounds, Calafat et al. (2005, 2008a, 2008b, 2008c) have shown that nearly all (BPA and BP-3) and three-quarters (triclosan) of free-living humans had measurable quantities of these chemicals in their urine.

In a recent cross-sectional study, Stahlhut et al. (2009) compared subjects who reported various lengths of fasting time and found that reported fasting time did not matter as much as expected when comparing longer fasting with shorter fasting subjects. There is a possibility that BPA may accumulate in body tissues. Because BPA is cleared by the kidneys, the pharmacokinetics of environmental phenols could be very different in populations with impaired renal function. In the present study, we evaluated the hypothesis that urinary excretion of BPA and APs may be reduced among people with insufficient renal function, as measured by estimated glomerular filtration rate (eGFR) using newly available data from the 2003–2006 National Health and Nutrition Examination Survey (NHANES).

## Materials and Methods

### Study population

We used data from the 2003–2006 NHANES for this analysis. NHANES, conducted by the Centers for Disease Control and Prevention (CDC), National Center for Health Statistics (NCHS), investigates a nationally representative sample using a multistage, stratified, clustered sampling strategy among the civilian, noninstitutionalized U.S. population. The 2003–2006 NHANES was reviewed and approved by the NCHS Institutional Review Board. Details on the methods used in the NHANES surveys are described elsewhere (CDC 2011).

In the 2003–2006 NHANES, a one-third subsample of participants ≥ 6 years of age (2,638 from the 2003–2004 NHANES and 2,612 from the 2005–2006 NHANES) was randomly selected for the assay of urinary excretion rate of BPA and APs; 185 participants with missing BPA and AP data and 953 participants with missing serum creatinine (Scr) or urinary creatinine (Scr was used to estimate kidney function) data were excluded. It is possible that people with known renal diseases may change their usual diet, which may in turn lead to alterations in levels of BPA and APs. Therefore, we excluded from our analysis 61 participants who reported that they had weak or failing kidneys and those who had received dialysis in the previous 12 months. Because history of renal diseases was collected only from those ≥ 20 years of age, we excluded 1,302 participants who were < 20 years of age. Also excluded were 176 pregnant or lactating females. As a result, 2,573 adults were included in the final analysis.

### Assessment of urinary BPA and APs concentrations

One spot urine sample was collected from participants in the 2003–2006 NHANES. Urinary BPA and APs were measured at the Division of Environmental Health Laboratory Sciences, National Center for Environmental Health, via online solid-phase extraction coupled with high-performance liquid chromatography/isotope-dilution and tandem mass spectrometry (CDC 2007, 2009). A comprehensive quality control mechanism (CDC 2007, 2009), including reagent blanks, was used to prevent contamination during urine specimen handling, storage, shipping, and analysis. For concentrations below the limit of detection (LOD), values equal to the LOD divided by the square root of 2 were used (Calafat et al. 2008a, 2008b, 2008c). According to the *Fourth National Report on Human Exposure to Environmental Chemicals*, the 2003–2004 data for tOP have been removed (CDC 2010). Thus, we did not include tOP in the present study.

### Measures of renal function

Of the 2003–2006 NHANES participants, > 92% donated a blood sample. Scr was measured using a kinetic rate Jaffe method. To appropriately calculate eGFR, all Scr measurements were recalibrated to standardized creatinine measurements obtained at the Cleveland Clinic Research Laboratory (Cleveland, OH, USA) following recommendations of the National Kidney Disease Education Program (Myers et al. 2006) and NHANES (Selvin et al. 2007). A recalibration equation was applied to Scr in the 2005–2006 survey (standardized creatinine = −0.016 + 0.978 × NHANES 2005–2006 uncalibrated Scr). No correction to the creatinine values in the 2003–2004 survey was needed (Selvin et al. 2007).

Scr-based eGFR was calculated using the modified four-variable Modification of Diet in Renal Disease (MDRD) Study equation (Levey et al. 2006) and the newly developed Chronic Kidney Disease Epidemiology Collaboration (CKD-EPI) equation (Levey et al. 2009):


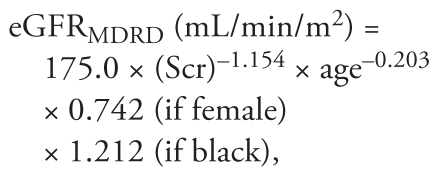


where age was expressed in years and Scr was standardized Scr level in milligrams per deciliter;


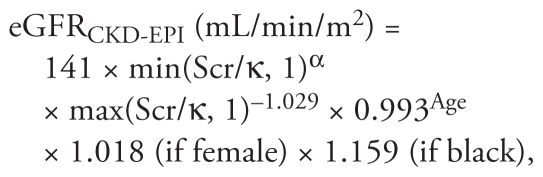


where Scr is standardized Scr, κ is 0.7 for females and 0.9 for males, α is −0.329 for females and −0.411 for males, min indicates the minimum of Scr/κ or 1, and max indicates the maximum of Scr/κ or 1.

We classified the participants into the following three categories: participants who had normal renal function with eGFR ≥ 90 mL/min/m^2^, participants who had mildly decreased renal function with eGFR ≥ 60 but < 90 mL/min/m^2^, and participants who had chronic kidney disease (CKD) with eGFR < 60 mL/min/m^2^ (National Kidney Foundation 2002).

### Covariates

Unadjusted urinary concentrations of BPA and APs (nanograms per milliliter) were used with urinary creatinine (milligrams per deciliter) adjusted in the model, according to previous recommendations (Barr et al. 2005). Several factors were evaluated for potential confounding and included in the statistical models: education level [less than high school diploma; high school diploma, including General Equivalent Diploma (GED); and at least some college education], annual household income (< $25,000, $25,000–55,000, > $55,000, and unclear), body mass index (BMI), waist circumference, dietary intake of energy, cigarette smoking status (nonsmoker, former smoker, and current smoker), alcohol drinking status (nondrinker, former drinker, and current drinker), and daily activities (sits/not walk very much, walk/not carry much, light load, and heavy work by incorporating accelerometer measures). Because both equations for eGFR include not only creatinine but also age, sex, and race, further adjustments for these three variables may lead to overadjustment. Thus, we present results without adjusting for these variables. However, we have conducted analyses by additionally adjusting for age, sex, and race. In the 2003–2006 NHANES, participants were also asked to provide self-reported data on physician diagnosis of several diseases and symptoms. In our study, cardiovascular disease (CVD) was defined as self-reported history of angina, coronary heart disease, heart attack, and shock. We calculated age-standardized CVD prevalence using the 2000 Census data for the U.S. population (U.S. Census Bureau 2007).

### Statistical analysis

We performed statistical analyses using the “Survey” procedure in SAS software (version 9.2; SAS Institute Inc., Cary, NC, USA) to estimate variance after incorporating the weights for the sample population, which take into account unequal selection probabilities and planned oversampling of certain subgroups that result from the complex multistage probability design in the NHANES.

To investigate the association between urinary excretion of BPA and APs, and renal function, we used regression models in the “Survey” procedure to calculate geometric means of urinary BPA and AP concentrations by eGFR category, after adjusting for potential confounding factors. Tests for trends were performed by entering the categorical variables as continuous variables in the model. All of the reported *p*-values were two-tailed, and statistical significance was set at 0.05.

## Results

Of the 2,573 subjects without known renal diseases, 1,074 (41.7%) had normal renal function, 1,231 (47.8%) had mildly decreased renal function, and 268 (10.4%) had undiagnosed CKD when we used the MDRD Study equation. The CKD-EPI equation led to a higher prevalence of normal renal function (57.4%) and a lower prevalence of mildly decreased renal function (33.1%). Tables 1 and 2 show baseline characteristics of participants by eGFR category estimated by using the two equations. Overall, participants with lower eGFR levels were more likely to be older, non-Hispanic white, former smokers, former alcohol drinkers, and physically inactive and to have had a lower educational attainment, lower income, a higher BMI, a higher waist circumference, and a lower dietary energy intake regardless of the equation. However, we observed significant differences in eGFR by sex only when we used the MDRD Study equation, and not the CKD-EPI equation. Compared with participants having normal renal function, participants with progressively impaired renal function had higher age-standardized CVD prevalence when we used the MDRD Study equation (6.37%, 8.12%, and 16.47%, respectively; *p* for trend < 0.01) or the CKD-EPI equation (6.07%, 9.31%, and 20.11%, respectively; *p* for trend < 0.01).

Table 3 presents the associations of renal function with urinary BPA and APs. The adjusted geometric means for urinary BPA excretion decreased with decreasing renal function using the MDRD Study equation, from 2.46 ng/mL for those with normal renal function to 2.31 ng/mL for those with mildly decreased renal function, and to 2.04 ng/mL for those with CKD (*p* for trend = 0.04). Although the trend of BPA excretion with eGFR was similar when we used the CKD-EPI equation, the test for trend was not statistically significant (*p* for trend = 0.41). We also found that the adjusted geometric means for urinary triclosan excretion decreased with decreasing levels of eGFR regardless of the equation (*p* for trend < 0.01 and 0.01, respectively, for MDRD Study and CKD-EPI equations). However, we did not find an association between urinary BP-3 and renal function (*p* for trend = 0.30 and 0.64, respectively). We also conducted analyses by including age, sex, and race in the models and found that the associations did not change substantially (data not shown).

To examine whether sex and age modify the association between renal function and excretion of environmental phenols, we conducted stratified analyses by sex (male, female) and age (≥ 65 years, < 65 years). The association between BPA excretion and renal function appeared primarily in females (*p* for trend = 0.03), and not in males (*p* for trend = 0.97), when we used the MDRD Study equation (Table 4). The test for interaction was not significant. When we used the CKD-EPI equation, the adjusted geometric means for urinary BPA excretion still decreased with decreasing renal function among females, from 1.90 ng/mL for those with normal renal function to 1.81 ng/mL for those with mildly decreased renal function, to 1.57 ng/mL for those with CKD, but the trend was not statistically significant (*p* for trend = 0.29). The average excretion for urinary triclosan decreased with reducing levels of eGFR among both males and females regardless of the equation, although the *p*-value for interaction was not statistically significant. We found no association between urinary BP-3 and renal function in males or females, although the BP-3 concentrations for females were significantly higher than those for males in each eGFR category. Urinary excretion of triclosan decreased with reducing renal function among younger participants (*p* for trend < 0.01 and 0.02, respectively, for MDRD Study and CKD-EPI equations) but not among older participants. The interactions between triclosan and age were statistically significant (*p*-value = 0.02 and 0.04, respectively, for MDRD Study and CKD-EPI equations).

## Discussion

In the present study, we found that persons without known renal diseases (*n* = 1,499 using the MDRD Study equation, and *n* = 1,096 using the CKD-EPI equation) had mildly decreased renal function or undiagnosed CKD. Urinary excretion levels of BPA significantly decreased with decreasing renal function when we used the MDRD Study equation, but the association was not significant when we used the CKD-EPI equation. Urinary excretion levels of triclosan decreased with decreasing renal function regardless of the equation. Conversely, we did not find a similar association for BP-3. For BPA, the association appeared primarily in females. For triclosan, the association appeared in both males and females. Furthermore, the association for triclosan primarily appeared among those < 65 years of age. To our knowledge, this is the first study to examine the associations between renal function and excretion levels of urinary environmental phenols.

Humans are exposed to a vast number of man-made chemicals, and the consequences of these exposures have not been clearly defined and are sometimes controversial (Turner and Sharpe 1997; Vandenberg et al. 2009). BPA and APs, particularly BPA, have been placed at the center of debate because of their phenol structure and hormone-like effects on the endocrine system and their adverse effects on human health (Amaral Mendes 2002). In the last decade, several epidemiological studies have investigated the relationship between BPA exposure and health-related end points, such as circulating sex hormone abnormities (Hanaoka et al. 2002), polycystic ovary syndrome (Takeuchi and Tsutsumi 2002; Takeuchi et al. 2004), fetuses with abnormal karyotype (Yamada et al. 2002), sterility (Kuroda et al. 2003), endometrial carcinoma and hyperplasia (Hiroi et al. 2004), obesity (Takeuchi et al. 2004), recurrent miscarriage (Sugiura-Ogasawara et al. 2005), and more recently, CVD, diabetes, and abnormal concentrations of liver enzymes (Lang et al. 2008; Melzer et al. 2010). In humans, it is generally understood that most ingested environmental phenols are rapidly conjugated with glucuronic acid in the gut wall and liver and almost completely excreted in urine as highly water-soluble metabolites with short terminal half-lives (Dekant and Völkel 2008; Ye et al. 2005). Because of sample abundance and noninvasiveness of urine collection compared with blood samples (Needham and Sexton 2000), most previous human studies have used total concentrations of BPA, tOP, BP-3, and triclosan in urine as biomarkers to assess exposure risk (Calafat et al. 2008a, 2008b, 2008c; Lang et al. 2008; Melzer et al. 2010; Ye et al. 2005). The kidneys are the major organs for the excretion of these environmental phenols. Renal excretion of xenobiotics is dependent on glomerular filtration rate, renal tubular secretion, and reabsorption. Some organic compounds, such as phenols, are glucuronidated in the liver and eliminated by active tubular secretion (Boeniger et al. 1993). In our study, we found that impaired renal function was associated with reduced excretion of triclosan, and perhaps BPA. The exact mechanism for the reduced excretion of some environmental phenols in CKD patients is not clear. Although accumulation of these environmental phenols is one possible explanation, it is also possible that these environmental phenols are metabolized elsewhere or circulated in an inactive form, such as conjugated phenols (Kanno et al. 2007; Murakami et al. 2007; Swan and Bennett 1992; Tett et al. 2003; Turnheim 1991).

CKD is a common and an increasing health threat among adults in the United States and worldwide (Coresh et al. 2007; Meguid El Nahas and Bello 2005). Recent U.S. CKD prevalence estimates indicate that 15.3% of the civilian noninstitutionalized population has CKD (Whaley-Connell et al. 2008). Compared with its high prevalence, the disease awareness among U.S. adults is generally low (Plantinga et al. 2008). It is well documented that patients with CKD have a high risk for premature death as a result of CVD compared with the general population (Foley et al. 1998). The mechanisms underlying this increased risk of CVD among CKD patients are not entirely clear (Stenvinkel et al. 2008). Two recent reports found a link between urinary BPA concentrations and prevalence of heart diseases using 2003–2006 NHANES data, suggesting an association between BPA exposure and CVD (Lang et al. 2008; Melzer et al. 2010). Previous studies suggest that conjugated BPA may be deconjugated into biologically active forms in some situations (Vandenberg et al. 2009). Future studies are necessary to investigate this issue among CKD patients or those with mildly decreased renal function. Importantly, in patients with end-stage renal disease on hemodialysis, in addition to almost complete loss of renal function and therefore loss of the ability to excrete environmental phenols, hemodializers may be an extra source for BPA accumulation in the body (Haishima et al. 2001; Yamasaki et al. 2001). Therefore, further studies are warranted to evaluate the associations between environmental phenols in the body and risk of CVD among those with CKD patients.

Based on Scr level, age, sex, and race, the MDRD Study equation is recommended in the National Kidney Foundation Kidney Disease Outcomes Quality Initiative classification guidelines and is the most widely used equation in the clinic, as well as epidemiological studies (Coresh et al. 2007; Levey et al. 2007). Its limitations include imprecision and systematic underestimation of measured glomerular filtration rate at higher values (Stevens et al. 2007). The newly developed CKD-EPI equation has been shown to overcome these limitations to some extent (Levey et al. 2009). In the present study, we conducted analyses using both equations. The CKD-EPI equation yielded a higher prevalence of normal renal function (57.4% vs. 41.7%) and a lower prevalence of mildly decreased renal function (33.1% vs. 47.8%) compared with rates generated with the MDRD Study equation. This finding is consistent with results from a recent report (Stevens et al. 2010). Furthermore, the significant difference in eGFR by sex and the significant association between urinary BPA and eGFR found using the MDRD Study equation became nonsignificant when we used the CKD-EPI equation. We cannot give an exact explanation for the different results from two equations because no measured glomerular filtration rate was available in the study. Therefore, future studies are needed to further investigate the association of urinary as well as blood BPA and other phenols with kidney function using measured glomerular filtration rate.

One interesting trend in the present study is that the differences in urinary BPA excretions between males and females increased with reduced eGFR categories regardless of the equation used. We observed reduced urinary levels of BPA with decreasing renal function only in females, and not in males, although this was significant only when we used the MDRD Study equation. The underlying mechanisms for the findings are unclear. Sex-associated pharmacokinetic and pharmacodynamic differences in phenol metabolism may explain at least part of these results (Schwartz 2003). Particularly, given that BPA has a structure similar to that of estrogens, it is conceivable that metabolism patterns could be different in males and females because of the different levels of enzymes related to estrogens and estrogen metabolism. On the other hand, the different associations by sex could be due to chance because we did not find a statistically significant interaction. These findings need to be replicated in future studies with larger sample sizes.

We observed no differences in urinary excretion of BP-3 by eGFR category. BP-3, also known as oxybenzone, is a monomethoxylated and monohydroxylated derivative of benzophenone. BP-3 is a principal component of ultraviolet filters as well as an indirect food additive (Gonzalez et al. 2006). The different results of BP-3 from BPA and triclosan in our study can probably be explained by its different chemical structure and physical and chemical properties.

This study has several strengths and implications. This is a population-based study with nationally representative samples. Participation rates for the interview and medical examination center during 2003–2006 NHANES were high, which may minimize potential selection biases. We excluded from analyses participants with self-reported kidney disease to minimize the influence of diet change on exposure. However, as with all prevalent case–control studies, one major concern is that the temporal sequence may not be clear. It is possible that the associations of urinary excretions of BPA and triclosan with renal function may be solely due to lower dietary intakes, and thus a lower exposure, among those with poorer renal function. However, we have adjusted for total energy intake and BMI as well as potential confounding factors in the model. Also, we did not find a similar association for urinary BP-3. Furthermore, we found a positive association between urinary BPA and renal function among females, but not among males, indicating that the association cannot be explained entirely by differences in dietary intake. In the present study, we used eGFR estimated by the MDRD Study equation and the CKD-EPI equation and found some different results, particularly for BPA. In the present study, we used eGFR estimated by the MDRD Study equation and the CKD-EPI equation and found different results, particularly for BPA. In addition, in our study we used the estimated eGFR as a measure of renal function, which is based on Scr. Therefore, future studies should use measured glomerular filtration rate. Because we used spot urine samples to estimate concentrations of BPA and APs, urine creatinine needs to be used to correct for urine dilution. However, the adjusted geometric means for urinary creatinine excretion significantly increased with decreasing renal function (*p* for trend < 0.01), from 91.71 mg/dL for those with normal renal function to 112.68 mg/dL for those with mildly decreased renal function, to 145.62 mg/dL for those with CKD using the MDRD Study equation, and we found the same significant trend when we used CKD-EPI equation with the corresponding geometric means of 94.29, 113.77, and 146.67 mg/dL, respectively (*p* for trend < 0.01). In other words, it is possible that even though BPA and APs are not actually associated with renal function, we may still find a correlation due to the association between Scr and urinary creatinine. Thus, we used uncorrected BPA and APs as dependent variables and included urinary creatinine as a covariate in regression models as recommended previously (Lang et al. 2008; Melzer et al. 2010). However, it is still possible that adjusting for urinary creatinine as a covariate may lead to potential bias, although the nature of bias is hard to predict, particularly if a larger proportion of creatinine in the urine of individuals with renal dysfunction is present because of pathways independent of glomerular filtration. In this regard, further studies measuring concentrations of BPA and APs in 24-hour urine samples are warranted to address this issue. Given the widespread human exposure to environmental phenols, future human studies of BPA and other environmental phenols should measure serum levels of these compounds in addition to urinary excretion levels. It is very important to understand the mechanisms by which the kidneys excrete environmental phenols, particularly in animal models of impaired renal function.

## Conclusion

Urinary excretion levels of triclosan, and possibly BPA, decreased with decreasing renal function. The associations might differ by age and/or sex. Further studies, particularly prospective studies with measurements from both serum levels and urinary excretions of environmental phenols, are warranted to evaluate the contribution of environmental phenols to the development of complications, including cardiovascular complication in CKD population.

## Figures and Tables

**Table 1:** Baseline demographic and selected risk factors by eGFR_MDRD_ category (*n* = 2,573).^a^

	eGFR_MDRD_, mL/min/1.73 m^2^			
Participant characteristic	≥ 90 (*n* = 1,074)	60–90 (*n* = 1,231)	< 60 (*n* = 268)	*p*-Value^b^
Male	559 (51.6)	648 (51.0)	129 (40.1)	0.0059
Age at screening (years)	38.0 (37.3–38.6)	49.8 (48.6–50.9)	71.4 (69.6–73.1)	< 0.0001
Race/ethnicity				< 0.0001
Mexican American	270 (12.0)	181 (4.2)	24 (1.5 )	
Other Hispanic	45 (5.1)	43 (3.1)	4 (1.3)	
Non-Hispanic white	392 (59.4)	775 (81.2)	197 (85.4)	
Non-Hispanic black	316 (16.6)	185 (6.7)	31 (5.9)	
Other race	51 (6.9)	47 (4.9)	12 (5.9)	
Educational attainment				< 0.0001
Less than high school diploma	322 (20.4)	278 (12.8)	94 (27.7)	
High school diploma (including GED)	259 (26.2)	298 (24.3)	68 (26.8)	
Some college or above	493 (53.4)	655 (62.8)	104 (45.5)	
Household income				< 0.0001
< $25,000	324 (24.4)	336 (18.7)	106 (36.4)	
$25,000–55,000	355 (33.4)	368 (29.2)	89 (38.4)	
> $55,000	339 (42.2)	458 (52.1)	45 (25.2)	
BMI (kg/m^2^)	27.7 (27.2–28.2)	28.6 (28.2–29.1)	29.2 (28.3–30.2)	0.0004
Waist circumference (cm)	94.9 (93.6–96.1)	98.4 (97.4–99.4)	102.4 (100.0–104.8)	< 0.0001
Dietary energy intake (kcal)	2333.2 (2255.9–2410.5)	2150.6 (2075.7–2225.6)	1632.4 (1537.5–1727.2)	< 0.0001
Cigarette smoking status				< 0.0001
Nonsmoker	549 (48.4)	620 (51.5)	132 (51.2)	
Former smoker	161 (15.0)	334 (25.3)	106 (37.9)	
Current smoker	363 (36.7)	274 (23.2)	29 (10.9)	
Alcohol drinking status				< 0.0001
Nondrinker	116 (10.0)	151 (10.2)	56 (23.0)	
Former drinker	173 (14.6)	220 (16.1)	85 (28.7)	
Current drinker	697 (75.5)	795 (73.7)	106 (48.3)	
Daily activities				< 0.0001
Sits/not walk very much	217 (20.6)	314 (25.6)	95 (29.0)	
Walk/not carry much	567 (51.7)	620 (47.9)	133 (52.7)	
Light load	181 (18.0)	226 (20.5)	33 (16.6)	
Heavy work	107 (9.8)	71 (6.1)	6 (1.6)	
CVD^c^	6.37 (0.71)^d^	8.12 (0.98)	16.47 (3.11)	< 0.0001

a Values are unweighted frequencies (weighted percentages, %) or weighted means (95% confidence intervals).

b Rao–Scott chi-square test for categorical data, and survey regression model for continuous variables.

c CVD includes angina, coronary heart disease, heart attack, and shock.

d Value is age-standardized prevalence rate (standard error).

**Table 2:** Baseline demographic and selected risk factors by eGFR_CKD-EPI_ category (*n* = 2,573).^a^

	eGFR_EPI-CKD_, mL/min/1.73 m^2^			
Participant characteristic	≥ 90 (*n* = 1,477)	60–90 (*n* = 851)	< 60 (*n* = 245)	*p*-Value^b^
Male	747 (49.8)	461 (52.7)	128 (44.9)	0.2105
Age at screening (years)	38.9 (38.1–39.7)	55.7 (54.0–57.4)	73.5 (71.7–75.3)	< 0.0001
Race/ethnicity				< 0.0001
Mexican American	344 (10.2)	111 (2.6)	20 (1.4 )	
Other Hispanic	66 (4.8)	22 (2.2)	4 (1.5)	
Non-Hispanic white	632 (65.6)	554 (83.5)	178 (84.8)	
Non-Hispanic black	364 (12.7)	134 (7.3)	34 (7.4)	
Other race	71 (6.6)	30 (4.4)	9 (4.8)	
Educational attainment				< 0.0001
Less than high school diploma	385 (16.9)	219 (14.4)	90 (30.8)	
High school diploma (including GED)	348 (25.3)	213 (24.5)	64 (29.2)	
Some college or above	744 (57.8)	419 (61.1)	89 (40.0)	
Household income				< 0.0001
< $25,000	413 (21.6)	257 (20.9)	96 (36.0)	
$25,000–55,000	479 (32.2)	249 (28.5)	84 (41.4)	
> $55,000	511 (46.2)	292 (50.5)	39 (22.6)	
BMI (kg/m^2^)	27.9 (27.5–28.4)	28.8 (28.4–29.2)	29.2 (28.3–30.1)	0.0031
Waist circumference (cm)	95.3 (94.2–96.5)	99.7 (98.7–100.8)	103.3 (101.1–105.5)	< 0.0001
Dietary energy intake (kcal)	2293.0 (2221.4–2364.6)	2091.4 (2017.9–2164.9)	1644.3 (1522.7–1765.8)	< 0.0001
Cigarette smoking status				< 0.0001
Nonsmoker	768 (50.1)	409 (50.2)	124 (52.4)	
Former smoker	215 (15.2)	290 (31.7)	96 (37.0)	
Current smoker	492 (34.7)	150 (18.1)	24 (10.6)	
Alcohol drinking status				< 0.0001
Nondrinker	160 (9.7)	110 (11.1)	53 (23.8)	
Former drinker	218 (13.4)	184 (19.5)	76 (29.5)	
Current drinker	994 (76.9)	508 (69.5)	96 (46.6)	
Daily activities				< 0.0001
Sits/not walk very much	301 (21.0)	231 (27.3)	94 (33.1)	
Walk/not carry much	762 (50.3)	438 (48.0)	120 (53.8)	
Light load	270 (19.5)	145 (19.9)	25 (12.2)	
Heavy work	142 (9.1)	37 (4.9)	5 (0.9)	
CVD^c^	6.07 (0.73)^d^	9.31 (1.44)	20.11 (3.25)	< 0.0001

a Values are unweighted frequency (weighted percentages, %) or weighted means (95% confidence intervals).

b Rao–Scott chi-square test for categorical data, and survey regression model for continuous variables.

c CVD includes angina, coronary heart disease, heart attack, and shock.

d Value is age-standardized prevalence rate (standard error).

**Table 3:** Adjusted geometric mean concentrations and 95% confidence intervals of the environmental phenols BPA and APs (ng/mL) by level of eGFR.

	eGFR_MDRD_, mL/min/1.73 m^2^				eGFR_CKD-EPI_, mL/min/1.73 m^2^			
Environmental phenol	≥ 90	60–90	< 60	*p*-Value for trend^a^	≥ 90	60–90	< 60	*p*-Value for trend^a^
BPA	2.46 (2.41–0.2.50)	2.31 (2.31–2.31)	2.04 (1.76–2.36)	0.04	2.37 (2.37–2.37)	2.31 (2.09–2.56)	2.22 (1.92–2.56)	0.41
BP-3	11.86 (11.86–11.86)	13.92 (13.92–13.92)	11.89 (8.43–16.77)	0.30	12.48 (12.48–12.48)	14.15 (14.15–14.15)	10.15 (6.64–15.53)	0.64
Triclosan	19.40 (13.66–27.56)	14.68 (11.17–19.29)	13.76 (8.62–21.98)	0.003	18.75 (13.36–26.31)	14.21 (10.04–20.12)	13.56 (8.21–22.41)	0.01

a Survey regression models were used after adjusting for educational attainment, household income, number of people in the household, BMI, waist circumference, smoking status, alcohol drinking, dietary energy intake, daily activities, CVD, and urinary creatinine.

**Table 4:** Adjusted geometric mean concentration and 95% confidence intervals of the environmental phenols BPA and APs by level of eGFR, stratified by sex or age.

Environmental phenol	eGFR_MDRD_, mL/min/1.73 m^2^					eGFR_CKD-EPI_, mL/min/1.73 m^2^				
	≥ 90	60–90	< 60	*p*-Value for trend^a^	*p*-Value for interaction	≥ 90	60–90	< 60	*p*-Value for trend^a^	*p*-Value for interaction
BPA										
Male	2.65 (2.65–2.65)	2.70 (2.70–2.70)	2.49 (2.00–3.10)	0.97	0.19	2.89 (2.40–3.47)	2.95 (2.32–3.76)	3.25 (2.27–4.65)	0.75	0.55
Female	2.06 (1.52–2.79)	1.77 (1.31–2.39)	1.52 (1.14–2.02)	0.03		1.90 (1.42–2.55)	1.81 (1.31–2.49)	1.57 (1.18–2.10)	0.29	
≥ 65 years	2.62 (1.45–4.72)	2.04 (1.42–2.93)	2.04 (1.40–2.97)	0.30	0.75	2.30 (1.34–3.94)	2.00 (1.38–2.90)	2.07 (1.43–3.00)	0.74	0.73
< 65 years	2.65 (2.35–2.99)	2.55 (2.32–2.79)	1.88 (1.16–3.04)	0.18		2.58 (2.40–2.77)	2.58 (2.17–3.07)	2.38 (1.38–4.09)	0.94	
BP-3										
Male	8.18 (8.18–8.18)	10.06 (10.06–10.06)	7.14 (4.99–10.21)	0.32	0.61	9.91 (6.39–15.37)	12.83 (7.47–22.01)	7.34 (3.35–16.07)	0.72	0.99
Female	25.10 (17.16–36.74)	27.13 (17.52–42.03)	20.76 (10.20–42.24)	0.94		25.24 (17.37–36.68)	26.22 (16.21–42.39)	19.90 (8.97–44.18)	0.80	
≥ 65 years	9.59 (3.26–28.19)	8.03 (3.48–18.51)	7.40 (2.73–20.11)	0.64	0.01	10.38 (5.19–20.76)	7.99 (3.22–19.80)	6.48 (2.39–17.59)	0.31	0.004
< 65 years	13.61 (12.31–15.05)	17.05 (14.52–20.01)	18.67 (12.09–28.83)	0.06		14.12 (12.84–15.53)	17.71 (17.71–17.71)	15.75 (6.41–38.71)	0.09	
Triclosan										
Male	23.05 (14.44–36.79)	17.85 (12.21–26.09)	14.42 (7.69–27.03)	0.07	0.89	23.84 (11.12–51.11)	21.25 (8.38–53.87)	14.51 (5.48–38.40)	0.14	0.66
Female	18.84 (9.01–39.98)	13.53 (6.91–26.49)	14.87 (6.91–32.02)	0.07		18.82 (9.18–38.59)	11.58 (5.94–22.55)	16.52 (6.52–41.87)	0.03	
≥ 65 years	9.92 (4.30–22.88)	5.74 (3.19–10.34)	7.41 (3.51–15.66)	0.91	0.02	12.17 (5.78–25.60)	5.42 (2.83–10.40)	6.23 (3.05–12.72)	0.19	0.04
< 65 years	21.74 (13.99–33.78)	16.80 (11.69–24.16)	8.45 (3.97–17.99)	0.008		20.88 (13.66–31.91)	15.98 (9.99–25.57)	7.98 (3.92–16.25)	0.02	

a Survey regression models were used after adjusting for educational attainment, household income, number of people in the household, BMI, waist circumference, smoking status, alcohol drinking, dietary energy intake, daily activities, CVD, and urinary creatinine.
